# Assessment of humoral immunity and nutritionally essential trace elements in steady-state sickle cell disease Nigerian children before and after *Prevenar 13* pneumococcal vaccination

**DOI:** 10.1097/BS9.0000000000000115

**Published:** 2022-07-06

**Authors:** Ganiyu Olatunbosun Arinola, Elizabeth Disu, Adedokun Babatunde, Christopher Olopade, Olufunmilayo Olopade

**Affiliations:** a Department of Immunology, College of Medicine, University of Ibadan, Nigeria; b Department of Pediatrics, Lagos State University Teaching Hospital, Lagos State, Nigeria; c Department of Medicine and the Center for Global Health, University of Chicago, Chicago, USA; d Center for Clinical Cancer Genetics and the Center for Global Health, University of Chicago, Chicago, USA.

**Keywords:** Immunoglobulin classes, Innate Immunity, *Prevenar* 13 pneumococcal vaccine, Sickle cell disease

## Abstract

Children with sickle cell disease (SCD) are particularly prone to pneumococcal infection and administration of *Prevenar* 13 pneumococcal vaccine in Nigerian children with SCD is yet to be wide spread. This call for the need to study humoral immune responses stimulated by *Prevenar 13* pneumococcal vaccine in SCD children to confirm the benefit or otherwise for the use of *Prevenar 13* pneumococcal vaccine.

**Method:**

The levels of humoral (innate and adaptive) immune factors and associated nutritionally essential trace elements were determined following *Prevenar 13* pneumococcal vaccination of 23 Nigerian children with SCD. Serum innate humoral immune factors [Complement factors (C1q and C4), transferrin, ferritin, and C-reactive protein (CRP)] and adaptive humoral immune factors [IgG, IgA, IgM, and IgE] were determined using ELISA. Nutritionally essential trace elements such as iron (Fe), copper (Cu), and zinc (Zn) were measured also using an atomic absorption spectrophotometer.

**Results:**

The serum levels of certain innate humoral immune factors (ferritin, CRP, and C4), only one adaptive humoral immune factors (IgE), and essential trace elements (Fe, Zn, and Cu) were significantly elevated in children with SCD post *Prevenar 13* pneumococcal vaccination when compared to prevaccination levels.

**Conclusion:**

Vaccination of children with SCD with *Prevenar 13* pneumococcal vaccine was associated with increased levels of more innate humoral immune factors than adaptive factors. This study thus supports the administration of *Prevenar 13* pneumococcal vaccination to children with SCD.

## 1. INTRODUCTION

Sickle cell disease (SCD) is an inherited autosomal recessive disorder in which there is abnormal hemoglobin (HbS) causing red blood cell sickling that damages the membrane of red blood cells (RBC) and decreases RBCs elasticity leading to hemolysis, anemia, vascular occlusion, and infarcts.^1^ In Africa, World Health Organization estimated that >300,000 babies are born with severe forms of hemoglobinopathies each year. Seventy-five percent of these babies are in sub-Saharan Africa, Nigeria accounts for 100,000 new births every year.^2^ One fifty thousand (150,000) children are born with SCD annually, representing the highest number of persons born with the SCD globally.^3,4^ Despite substantial morbidity and mortality associated with SCD among Nigerian children, Inusa et al^5^ reported no established interventional programs to prevent death of SCD. Also SCD forms a small part of clinical practice of general duty doctors.^6^ This is despite of the attention drawn to the importance of developing a concrete programme by Ngwube et al^7^ and realization that the morbidity and mortality of children with SCD in Nigeria are preventable.^6–8^

The reason for relatively higher morbidity and mortality among children with SCD is complex and had been related to defective immune functions such as impaired phagocytosis,^9^ reduced C4 and C3, increased C1 inhibitor, reduced C3 activator,^10^ defective neutrophil migration/chemotactic activity and lymphocyte transformation,^11^ and inadequate antibody production due to functional asplenia, which make them susceptible to infections with encapsulated organisms such as *Escherichia coli*, *Streptococcus pneumonia*, and *Salmonella species.*^12–16^

Intracellular killing process during phagocytosis has also been reported to be impaired in patients with SCD,^9,11^ making SCD patients prone to infections caused by *S. pneumoniae* and *Haemophilus influenza.*^17–19^

Nutritionally essential trace metals (zinc [Zn], iron [Fe], selenium [Se], and copper [Cu]) are not antioxidants, but are integral parts of the antioxidant defense system and are necessary for the proper function of antioxidant enzymes like Fe-containing catalase, Se-glutathione peroxidase, and Cu/Zn-containing superoxide dismutase.^20^ Thus, it is likely that the levels of these nutritionally essential trace metals, which are important for proper functioning of antioxidant enzymes, might be affected SCD. Olaniyi and Arinola^21^ showed that selenium was reduced in HbSS patients compared with HbAA children and also highlighted that nutritional supplementation with certain essential trace elements will benefit SCD Nigerian patients. In a previous study, serum IgG and IgE concentrations was significant higher while serum levels of IgM and IgA were not statistically different in HbSS children compared with the HbAA group.^22^

Pneumococcal infections are a major cause of morbidity and mortality worldwide and pneumonia is the most common cause of pneumococcal-attributed death. Each year, an estimated one million children under 5 years of age die due to *S. pneumoniae* respiratory infections, with a disproportionate number of deaths occurring in developing countries.^23,24^ Pneumonia is one of the most common causes of morbidity and mortality in infancy and childhood in Nigeria.^12,13^ The use of penicillin prophylaxis against bacteria pathogens and pneumococcal infection with pneumococcal vaccination is widely accepted as the standard of care for patients with SCD in developed countries.^23–25^ Unfortunately, the administration of penicillin prophylaxis and pneumococcal vaccination is not widely adopted or affordable in Nigeria and most countries in sub-Saharan Africa.

Summarily, the literature showed inadequacies of certain aspects of immune responses in SCD Nigerian children compared with HbAA children. Since capacity for phagocytosis (innate immune response) and antibody levels (adaptive immune response) are parameters used to monitor immune responses to vaccination,^26^ this pilot study measured innate humoral immune factors [catalase, hydrogen peroxide, myeloperoxidase, superoxide dismutase, Complement factors (C1q and C4), transferrin, ferritin, and C-reactive protein], adaptive humoral immune factors (Immunoglobulin classes such as IgG, IgA, IgM, and IgE), and nutritionally essential trace elements such as iron (Fe), copper (Cu) and zinc (Zn) to determine the mechanism of protection stimulated by pneumococcal-conjugated vaccine (*Prevenar 13*) (Pfizer Inc, New York) in Nigerian children with SCD.

## 2. RESULTS

Table 1 shows that selected red cell indices of the HbSS children were normal and they were not experiencing crisis. Table 2 shows that levels of SOD (*P* = 0.023), catalase (*P* = 0.002), H_2_O_2_ (*P* = 0.007), and myeloperoxidase (*P* = 0.001) were significantly raised in children with SCD postvaccination when compared with prevaccination levels. Also in Table 2, ferritin (*P* = 0.001), CRP (*P* = 0.050), and C4 (*P* = 0.023) were significantly raised postvaccination compared with prevaccination. Among the Ig classes considered, only serum IgE level was significantly raised in SCD children postvaccination compared with prevaccination levels (*P* = 0.050) (Table 3). Essential trace elements (Fe [*P* = 0.001], Zn [*P* = 0.001], and Cu [*P* = 0.001]) were significantly elevated postvaccination compared with prevaccination levels (Table 4).

**Table 1:** Age, red cell indices and clinical symptoms of HbSS children.

Age	10–48 mo
Red cell indices	
Red cell count	3.5 ± 1.1
Hemoglobin concentration (g/dL)	6.9 ± 1.6
Hematocrit (%)	24.0 ± 5.3
Mean cell volume (fl)	73 ± 16.4
Mean corpuscular hemoglobin (pg)	21.7 ± 1.3
Mean cell hemoglobin concentration (g/dL)	28.6 ± 1.2
Clinical characteristics:	
Blood transfusion in the last 1 y	Nil
Painful crisis	Nil
Infection	Nil
Chronic renal failure	Nil
Systemic arterial hypertension	Nil
Splenectomy	Nil
Chronic respiratory disease	Nil

**Table 2:** Comparison of serum innate humoral immune factors in children with SCD pre- and postvaccination with conjugate pneumococcal vaccine.

Innate humoral factors (unit)	Normal range	Prevaccination, mean (SD)	Postvaccination, mean (SD)	*P* values*
C-reactive protein(mg/dL)	Below 3	4.4 (2.6)	6.9 (6.1)	0.050*
Ferritin (ng/dL)	12–300	96.3 (17.8)	197.9 (43.8)	0.001*
Transferrin (mg/dL)	204–360	158.9 (80.4)	268.2 (179)	0.073
C4 (g/mL)	10–50	21.4 (8.2)	33.8 (20.7)	0.023*
C1q (mg/dL)	10–25	28.8 (34.5)	28.8 (34.6)	0.309

* Statistically significant based on paired t tests.

**Table 3:** Comparison of serum levels of immunoglobulin classes in children with SCD pre- and postvaccination with conjugate pneumococcal vaccine.

Immunoglobulin classes (unit)	Normal range	Prevaccination, mean (SD)	Postvaccination, mean (SD)	*P* values*
IgG (mg/dL)	800–1800	950 (630)	743 (437)	0.159
IgA (mg/dL)	70–560	261 (137)	295 (136)	0.355
IgM (mg/dL)	54–220	45.9 (21.3)	38.8 (20.9)	0.219
IgE (iU/mL)	<100	166 (243)	368 (415)	0.050*

* Statistically significant based on paired t tests.

**Table 4:** Comparison of serum levels of nutritionally essential trace elements in children with SCD pre- and postvaccination with conjugate pneumococcal vaccine.

Essential trace elements (unit)	Normal range	Prevaccination, mean (SD)	Postvaccination, mean (SD)	*P* values*
Fe (μg/dL)	40–175	120.6 (30.4)	160.2 (13.7)	0.001*
Zn (μg/dL)	60–130	71.1 (8.3)	105 (7.2)	0.001*
Cu (μg/ml)	150–600	123.3 (16.9)	161.8 (20.6)	0.001*

* Statistically significant based on paired t tests.

## 3. DISCUSSION

Previous studies have suggested that patients with SCD may be unable to develop normal protective immune responses to some forms of antigenic challenge, especially vaccines.^27,29–31^ Overwhelming postsplenectomy infection (OPSI) by encapsulated bacteria such as *S. pneumoniae*, which accounts for over 70% of all infections, is a major health problem.^32^ We investigated responses of the immune system in children with SCD to pneumococcal polysaccharide vaccine. As shown in Table 1, the children with SCD were in steady state and not experiencing any adverse clinical condition. Our results also demonstrated that children with SCD produced adequate levels of IgG, IgA, IgM but increased levels of CRP, ferritin, C4, Fe, Zn, and Cu after pneumococcal vaccination. Our previous report showed that C-reactive protein was raised in HbSS, while the levels of tranferrin and haptoglobin were lowered in HbSS compared with HbAA^33^ in the absence of vaccination.

Pneumococcal infection is most devastating in settings of functional asplenia such as commonly seen in SCD.^34^ Similarly, deficiencies of complement factors contribute to inefficient phagocytosis and increased susceptibility to pneumococcal infections.^35^ Protective immunity, which is stimulated following pneumococcal vaccination, is usually initiated by stimulation of capsular polysaccharide (PS) antigens.^23^ Antibodies (Abs) to the PS capsule fix Complement factors to enable opsonization of *Pneumococci* for phagocytosis^23^. IgM is more effective in fixing complement factors and enhancing opsonization than IgG^36^. Therefore, reduced IgG and IgM are expected in SCD postvaccination as we observed in the present study. This observation is consistent with previous reports supporting the concept that PSs are usually poorly degradable, mostly stimulate mature B cells and often do not elicit responses in neonates.^37^ But increases of serum IgA and IgE in SCD children postvaccination might suggest that not only IgM memory B cells are solely responsible for anti-PS antibody production. Previous studies showed that sequence analysis of antipneumococcal PS Abs, 5 days postvaccination; demonstrated a predominance of IgG and IgA, which are presumably derived from switched memory cells that have undergone somatic hypermutation.^37,38^ Also in mice, the B1b cell subset participated in IgM and isotype-switched antibody production in response to pneumococcal PS.^39^ Raised IgE level in children with SCD postvaccination might be an indication of continuous inflammation in children with SCD with *Prevenar* pneumococcal vaccine. This is in support of raised other inflammatory factors (CRP and C4) in children with SCD postvaccination as observed in the present study.

To combat infections, immune cells use NADPH oxidase to reduce O_2_ to oxygen-free radical and H_2_O_2_. Neutrophils and monocytes utilize myeloperoxidase (MPO) to further combine H_2_O_2_ with Cl^−^ to produce hypochlorite, which plays a role in destroying ingested bacteria. The absence of MPO will prevent the formation of hypochlorite and will result in susceptibility to infections from intracellular organisms.^40^ Amer and Fibach^17^ reported that sickle cells generate about 2-fold greater quantities of superoxide, H_2_O_2_, and hydroxide (OH) than HbAA. Catalase is a common enzyme found in nearly all living organisms exposed to oxygen. It catalyzes the decomposition of hydrogen peroxide to water and oxygen. It is also a very important enzyme in protecting the cell from oxidative damage by reactive oxygen species. Cu, Zn and Fe are cofactors of superoxide dismutase (SOD) and catalase; thus, raised levels of these essential trace elements might be a response to increased need of antioxidant enzymes. Free iron is toxic to cells as it acts as a catalyst in the formation of free radicals from reactive oxygen species via the Fenton reaction.^41^ Microorganisms also depend on the presence of free iron to multiply. Ferritin is a ubiquitous intracellular protein that stores iron and releases it in a controlled fashion. The amount of ferritin stored reflects the amount of iron stored. Hence, raised levels of ferritin in our subjects postvaccination might be a protective measure to remove excess iron from circulation.

Inflammation is an innate immune response^42,43^ against harmful antigens.^44^ The observation of raised serum levels of C4, CRP, and ferritin suggested continuous inflammation in children with SCD postvaccination. Previous studies showed that SCD is inflammatory in nature.^29–33^

## 4. CONCLUSION

Vaccination of SCD children with *Prevenar* 13 pneumococcal vaccine was associated with increased levels of most innate humoral immune factors. This study thus supports the administration of *Prevenar* 13 pneumococcal vaccination to children with SCD. However, larger sample size and community-based study will give more power to the findings.

## 5. MATERIAL AND METHODS

The study was a longitudinal study conducted among children with SCD aged 5 to 60 months (5–60 months) attending the sickle cell clinic in 5 Lagos State Government Public Hospitals in Nigeria. Approval for the study was obtained from the Health Research and Ethics Committee of the Lagos State University Teaching Hospital. Hemoglobin electrophoresis was carried out to confirm genotype of all subjects. Twenty-three children with SCD whose parents gave consent were recruited. The children with SCD were brought for routine follow-up clinic evaluation and were in their steady state of health. Blood samples were collected on the day of vaccination of children with SCD with a dose of pneumococcal-conjugated vaccine (*Prevenar 13*) and at 1 month postvaccination. A sample of 5 mL of venous blood was collected from the ante cubital vein without venous stasis from each subject into a clean bottle without anticoagulant and allowed to clot at room temperature. The clotted blood in the plain serum bottle was allowed to retract; the serum was separated by centrifugation at 5000 rpm for 20 minutes.

### 5.1. Determination of IgG, IgA, IgM, IgE, ferritin, transferrin, C4, CRP, and C1q

Enzyme-linked immunoabsorbent assay was used to determine the levels of above parameters in the serum as previously described.^22^ The assay system utilizes 2 unique antibodies (a mouse monoclonal capture antibody and a goat polyclonal detection antibody) directed against epitope on individual immunoglobulin molecule. Plastic wells were coated with anti-Ig class (mouse monoclonal), and then test samples/controls containing Ig classes were added to the wells to form immune complexes. Anti-Ig class (goat polyclonal) enzyme-labeled with horseradish peroxidase was added to each well and incubated for 45 minutes at room temperature. The Ig molecule was sandwiched between the solid phase and enzyme-labeled antibodies. The samples were decanted and washed 5 times to remove unbound-labeled antibody. An enzyme chromogen was added to the wells and incubated for 15 minutes at room temperature, resulting in the development of a color. A stopper was added to each well to stop the reactions. The intensity of the color was directly proportional to the concentration of Ig in the sample.

### 5.2. Trace element determination

Serum levels of Fe, Zn, and Cu were determined using atomic absorption spectrophotometer as previously described.^28^

### 5.3. Statistical analysis

The result was presented in mean ± standard deviation. The differences between the means were determined using Student *t* test. The data was analyzed using Statistical Package for Social Science (SPSS) version 17.0. Level of significance was set at *P* ≤ 0.05.
