# Erratum: Assessment of humoral immunity and nutritionally essential trace elements in steady-state sickle cell disease Nigerian children before and after Prevenar 13 pneumococcal vaccination

**DOI:** 10.1097/BS9.0000000000000146

**Published:** 2023-01-18

In the article titled “*Assessment of humoral immunity and nutritionally essential trace elements in steady-state sickle cell disease Nigerian children before and after Prevenar 13 pneumococcal vaccination*,” published on pages 170-173, Issue 3, Volume 4 of Blood Science,^1^ the **Ethical Consideration statement** “Approval for the study was obtained from the Health Research and Ethics Committee of the Lagos State University Teaching Hospital and the approval number is LREC/10/06/454.” was omitted. The Journal and the authors wish to apologize for this error.
